# Standard vs. enhanced implementation strategies to increase adoption of a multidrug-resistant organism alert tool: a cluster randomized trial

**DOI:** 10.3389/frhs.2025.1566454

**Published:** 2025-09-18

**Authors:** Cara Ray, Cassie Goedken, Ashley M. Hughes, Geneva M. Wilson, Natalie R. Hicks, Margaret A. Fitzpatrick, Makoto M. Jones, Christopher Pfeiffer, James Stacey Klutts, Martin E. Evans, Katie Joy Suda, Charlesnika T. Evans

**Affiliations:** ^1^ Center of Innovation for Complex Chronic Healthcare, Edward Hines Jr. VA Hospital, Hines, IL, United States; ^2^ Iowa City VA Medical Center, Center for Access and Delivery Research and Evaluation, United States Department of Veterans Affairs, Iowa City, IA, United States; ^3^ Department of Medicine, School of Medicine, Case Western Reserve University, Cleveland, OH, United States; ^4^ Feinberg School of Medicine, Northwestern University, Chicago, IL, United States; ^5^ VA National Infectious Diseases Service, MDRO Prevention Division, Washington, DC, United States; ^6^ Rocky Mountain Regional VA Medical Center, Center of Innovation for Veteran Centered and Value Driven Care, Aurora, CO, United States; ^7^ Department of Medicine, University of Colorado Anschutz Medical Campus, Aurora, CO, United States; ^8^ VA Salt Lake City Health Care System, Informatics, Decision Enhancement, and Analytics Sciences Center, Salt Lake City, UT, United States; ^9^ Department of Internal Medicine, Division of Epidemiology, Spencer Fox Eccles School of Medicine, University of Utah, Salt Lake City, UT, United States; ^10^ Medicine Service, VA Portland Healthcare System, Portland, OR, United States; ^11^ Department of Medicine, School of Medicine, Oregon Health and Science University, Portland, OR, United States; ^12^ VA Central Office, VA National Pathology and Laboratory Medicine Program Office, Washington, DC, United States; ^13^ Iowa City VA Medical Center, Iowa City VA Health Care System, Veterans Health Administration, United States Department of Veterans Affairs, Iowa City, IA, United States; ^14^ Carver College of Medicine, Pittsburgh VA Medical Center, The University of Iowa, Iowa City, IA, United States; ^15^ Center for Health Equity Research and Promotion, Pittsburgh, PA, United States; ^16^ Division of General Internal Medicine, University of Pittsburgh, Pittsburgh, PA, United States

**Keywords:** infection prevention, implementation facilitation, user-centered design, veterans affairs, multidrug-resistant organism (MDRO)

## Abstract

**Background:**

The Veterans Health Administration (VHA) launched VA Bug Alert (VABA) to identify admitted patients who are infected or colonized with multidrug-resistant organisms (MDROs) in real time and promote timely infection prevention measures. However, initial VABA adoption was suboptimal. The objective of this project was to compare the effectiveness of standard vs. enhanced implementation strategies for improving VABA adoption.

**Methods:**

121 VA healthcare facilities were evaluated for adoption of VABA (at least 1 user registered at a facility) April 2021–September 2022. All facilities initially received standard implementation, which included: VABA revisions based on end-user feedback, education, and internal facilitation via monthly meetings with the MDRO Prevention Division of the VHA National Infectious Diseases Service. Surveys evaluated VABA perspectives among MDRO Prevention Coordinators (MPCs) and/or Infection Preventionists (IPs) before and after initial standard implementation. Facilities not registered for VABA following initial standard implementation (*n* = 31) were cluster-randomized to continue to receive standard implementation or enhanced implementation (audit and feedback reports and external facilitation via guided interviews to assess VABA use barriers). Percentages of facilities adopting VABA at baseline, after standard implementation (Follow-up 1), and after the enhanced vs. standard implementation trial period (Follow-up 2) were assessed and compared across time points using McNemar’s test. VABA adoption was compared by trial condition using Fisher's exact test.

**Results:**

Before education, 25% of 167 MPC/IP survey respondents across 116 facilities reported no knowledge/use of VABA. After education, 82% of 92 survey respondents across 80 facilities reported intending to use VABA. At baseline, VABA registrations were 40%. Registrations significantly increased aft Follow-up 1(75%, *p* < 0.01) and at Follow-up 2 (89%, *p* < 0.01). Adoption did not significantly differ by assigned implementation condition but was higher among facilities that completed all components of enhanced implementation than those who did not (87.5% vs. 43.5%, *p* = 0.045). Guided interviews revealed key facilitators of VABA registration, which included perceived fit, implementation activities, and organizational context (e.g., staffing resources).

**Conclusions:**

Implementation efforts dramatically increased VABA registrations. Incorporating interview feedback to increase VABA's fit with users' needs may increase its use and help reduce MDRO spread in VA.

## Background

Antimicrobial-resistant infections are a critical threat to public health. They cause three million illnesses, 48,000 deaths and $35 billion in excess costs each year in the United States (U.S.) (1). Multidrug-resistant organisms (MDROs) such as carbapenem-resistant *Enterobacterales* (CRE) are especially important. They are designated by the Centers for Disease Control and Prevention (CDC) as urgent threats to public health in the U.S (1). Patients who are infected/colonized with MDROs may transmit MDROs to others.

Identification and placement into isolation/contact precautions of such patients is recommended by the CDC and World Health Organization (WHO) (2, 3). Early identification is important to ensure appropriate precautions are enacted as soon as possible, minimizing the opportunity for MDRO transmission. However, actionable information regarding whether patients are colonized or infected with MDRO(s) from prior hospital admissions is often not readily available (4, 5). This information gap can delay isolation (4) and increase risk of MDRO spread. Therefore, such delays can contribute to MDRO outbreaks in hospitals.

The U.S. Veterans Health Administration (VHA) launched a tool to expedite identification of patients with a history of MDRO infection/colonization (6). This tool is now called VA Bug Alert (VABA) (6). VHA is a large, nationwide, integrated healthcare system. It uses a centralized electronic health record. This allows VHA to track and monitor MDRO-positive patients longitudinally and between hospitals system-wide. VABA alerts local facilities when such patients are admitted to their facility. VABA allows for more timely identification of MDRO-positive patients upon interfacility transfer than previous protocols involving manual tracking. Similar system-wide electronic alerts have been found to improve isolation precaution compliance (4, 7). Guidelines recommend such alerts for preventing MRSA infection and transmission (8).

Development and implementation of the tool was overseen by the MDRO Prevention Division of the VHA's National Infectious Diseases Service. Prior efforts to implement VABA included education provided to VA MDRO prevention coordinators (MPCs) and infection preventionists (IPs) nationwide in June 2020. The MDRO Prevention Division conducted an unpublished survey of 163 MPCs and/or IPs (response rate = 100%) in May 2021. It found that only 32.5% of survey participants were registered users of VABA (unpublished data). A lack of knowledge about its existence (62.7%) and how to register for it (37.3%) were reported reasons for non-use. Among those that did report using VABA, only 30.2% were routine users. Users indicated that including more organisms (69.8%) and the specimen source (54.7%) in VABA would make it more useful and increase utilization.

The MDRO Prevention Division and VABA developers launched a quality improvement initiative. The goal of this initiative was to improve VABA adoption and ultimately achieve VA-wide implementation. For this initiative, they partnered with the VA Combating Antimicrobial Resistance through Rapid Implementation of Guidelines and Evidence (CARRIAGE) II Quality Enhancement Research Initiative Program. This partnership is collectively henceforth called the VABA Group.

To accomplish the goals of the initiative, the VABA Group employed an adaptive protocol (9, 10). This protocol comprised strategies that were previously successful for implementing evidence-based practice interventions. Strategies delivered to all sites included facilitation from the MDRO Prevention Division (i.e., internal facilitation), education, and tailoring and adaptation (11–25). It was anticipated that some sites would not register shortly after deployment of these initial “standard” implementation strategies. For such sites, the CARRIAGE II team also provided external facilitation and audit and feedback (15, 26, 27). These “enhanced” implementation strategies are useful with late adopters (10, 25, 28, 29).

The current paper aims to describe these implementation efforts and their evaluation. We assessed the effects of implementation strategies on VABA adoption (i.e., registration) over time. We also compared the impact of enhanced vs. continued standard implementation on late adopters. We describe how target user perspectives were evaluated at multiple periods. These perspectives were used to refine and individualize implementation activities at subsequent periods. Implications for implementation science and future directions are discussed.

## Methods

### Ethical considerations for human subjects

This project was conducted as part of the CARRIAGE II QUERI program. This program was designated as a non-research quality improvement project for program implementation and evaluation purposes in accordance with VHA Program Guide 1200.21 the VHA National Infectious Diseases Service. Participants' decision to participate in surveys was taken as an indication of their consent. Verbal consent was obtained for participation in and audio recording of guided interviews.

### Study design and setting

**Figure 1:**
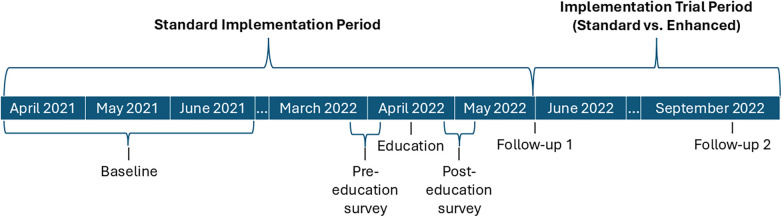
Implementation timeline. Timeline of implementation activities and registration (adoption) outcome measurement.

VA facilities were eligible for VABA use if they had an MPC, saw inpatients, and did not transition to an electronic health record vendor that was incompatible with VABA at the time. These facilities included stand-alone, single-hospital facilities and integrated, multi-hospital facilities. Eligible facilities (*n* = 121) and individual MPCs and IPs were evaluated for VABA registration and VABA beliefs and use using a pre-post design. Study procedures occurred March 2021 through October 2022. A timeline of implementation and measurement time periods can be found in Figure 1. Briefly, standard implementation was delivered to all sites in the Standard Implementation Period. Adoption was measured at baseline and Follow-up 1 (after the Standard Implementation Period). Facilities unregistered by Follow-up 1 underwent a cluster-randomized controlled trial to evaluate the impact of standard vs. enhanced implementation in the Implementation Trial Period on registration at Follow-up 2. Guided interviews were conducted with enhanced implementation facilities to assess barriers to VABA use. The Consolidated Standards of Reporting Trials were used to describe trial design.

### Intervention

The Template for Intervention Description and Replication checklist was used to compile this description of the intervention (30). VABA was originally developed as an alert system and web report for individuals for patients with CRE (6). The goal of this system was to target interfacility movement and transfers between all VA facilities. This strategy is a key measure to prevent transmission of MDROs. VABA's development has been described previously (6).

Briefly, VABA includes cases of MRSA, vancomycin-resistant *Enterococcus* (VRE)*,* and other organisms. VABA uses information from the VA Corporate Data Warehouse, a database of VA-wide patient-level health characteristics and care utilization (31). It provides automatic (push) email alerts for new admissions with prior positive results for these organisms (within the past 1 year for MRSA, and no time limit for other included organisms) and current admissions with new positive results. Alert settings can be customized to notify users about specific pathogens and culture result source facility (internal or external to the target user's facility). VABA also allows users to pull and sort reports of MDRO-positive admissions and recent weekend discharges, with information on positive surveillance swabs and clinical cultures for report entry. The target users for VABA are MPCs and IPs who are responsible for monitoring, tracking and reporting MDROs in VA.

### Implementation strategies

Implementation strategies are described in detail in Table 1. Standard strategies included: (1) tailoring of VABA to include new features based on target end-user feedback, (2) development and delivery of a VABA educational module to target users, and (3) ongoing internal facilitation by the MDRO Prevention Division. Enhanced implementation strategies included standard strategies plus: (1) audit and feedback reports on VABA registration (see Supplementary Figure S1) and (2) external facilitation from members of the CARRIAGE II team.

**Table 1:** VA bug alert implementation strategies.

Strategy	Source	Description	Dates
Standard (delivered to known MPCs/IPs at all eligible facilities)			
Tailoring based on target user feedback	VABA Developers, MDRO Prevention Division	• New name (VABA) • Ability to track additional organisms [namely, vancomycin-resistant *Enterococci* (VRE)] • Add info on MDRO-positive specimen type and originating facility (e.g., urine, nares).	April 2021–January 2022
Internal facilitation	MDRO Prevention Division & VABA Developers	• Sharing reminders, updates, and responses to questions/troubleshooting requests from the field through ongoing monthly national MPC calls • Maintaining storage of and access to the educational materials through the MDRO Prevention sharepoint facility • Monitoring VABA registrations and use	January 2022–October 2022
Educational module development	VABA Group	• History and rationale for development • Key features • How to use • How to register	January 2022–April 2022
Educational module delivery	MDRO Prevention Division	• Presentation of the educational module described in the above table cell on national VHA IP/MPC calls	April 2022
Enhanced (delivered along with standard implementation to MPCs/IPs at a random subset of facilities not registered by June 2022)			
Audit and feedback reports	CARRIAGE II Team (with development input from whole VABA Group)	• Percentage of eligible facilities registered for VABA • Statement that the recipient's facility was not registered • Information about VABA’s features • Notification of upcoming invitation to receive external facilitation	June 2022
External facilitation	CARRIAGE II Team (with procedure development input from the VABA group)	• Attending the monthly MPC calls to document VABA implementation activities and progress • Guided interviews about perspectives on VABA w/ real-time individualized VABA coaching via Microsoft Teams • Emails with information about VABA email, including sign-up instructions, education slides, contact information for the VABA Support Team, and responses to guided interview participants’ questions about VABA (e.g., how to sign up for CRE alerts) as needed	July 2022–August 2022

MPC, multidrug-resistant organism prevention coordinator; IP, infection preventionist.

External facilitation included guided, semi-structured interviews held via Microsoft Teams (MS) Teams with at least one MPC/IP from a target facility. These guided interviews were facilitation sessions where individualized, real-time VABA coaching was offered and provided to those who accepted the offer. Interviewers first assessed participants' knowledge, attitudes, and beliefs regarding VABA use. Information about how VABA may improve the ability to meet participants' MDRO prevention needs was offered to guided interview participants. Participants could choose to accept or decline to receive this additional information. For participants who accepted, this information was tailored in real-time to individual participants based on their post-education-survey-reported reasons for not registering for VABA or their specific needs noted in the guided interview. An example included showing participants how to obtain close-to-real-time admission notifications of patients whose MDROs were identified at VA facilities besides the end user's (a key feature of VABA).

Within the same guided interview, participants were also asked for their impression of the information shared (if they accepted additional information), VABA registration and use intentions, and recommendations for improving VABA and/or its' implementation efforts. External facilitators had backgrounds in implementation science, social psychology, social work, and epidemiology. They received basic training about VABA from the developers and MDRO Prevention Division. The VABA group met quarterly to share updates and preliminary results regarding VABA adoption and implementation (e.g., recommendations from the CARRIAGE II Team for VABA design and internal facilitation based on guided interview results, with additional meetings scheduled as needed.

### Randomization

**Figure 2:**
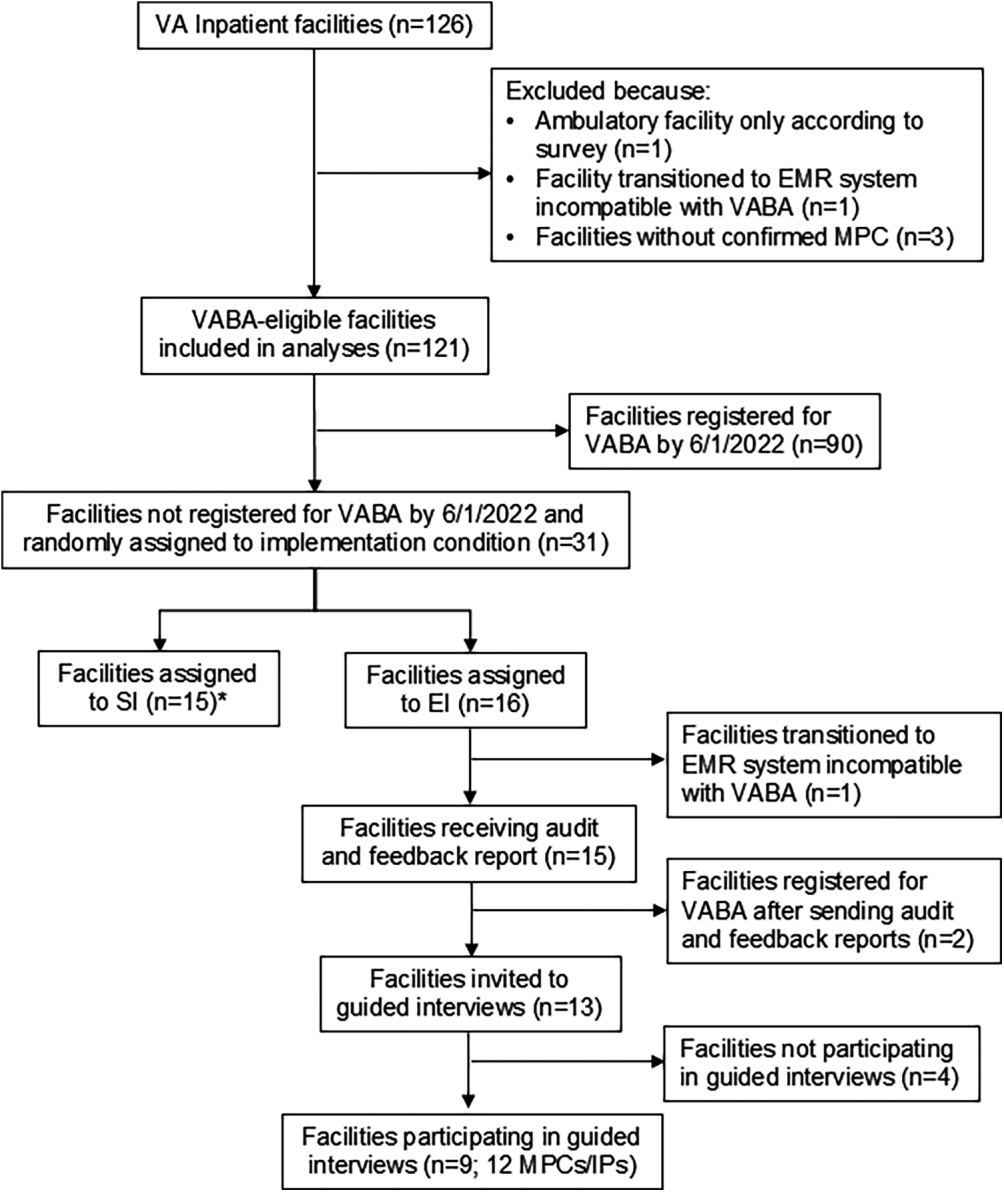
Registration analyses cohort creation/inclusion-exclusion diagram *Facilities that transferred to a new electronic health record system were identified and excluded from enhanced implementation after random assignment to implementation condition; only those that were included in the random assignment (*n* = 2, one in the enhanced implementation condition, one in the standard implementation condition) are included in analyses.

All facilities initially received standard implementation (see Figure 2 for cohort inclusion). If a facility was confirmed to have an MPC according to the MDRO Prevention Division and did not register by Follow-up 1 (*n* = 31), the facility was randomized to continue standard implementation alone (*n* = 15) or to enhanced implementation (*n* = 16). Random assignment to condition was performed by a member of the implementation team (C.R.) by entering VA facility codes into an online random assignment tool (32) and assigning them to standard vs. enhanced. The CARRIAGE III implementation team was not blinded to condition, as they only provided enhanced implementation to sites in the enhanced implementation condition. C.R. conducted the analyses unblinded to condition.

### Outcomes

The primary outcome measure of adoption was VABA registrations at the 121 eligible VA facilities. Registration was defined by having at least one user register at a given facility, as many VA facilities may only have 1 MPC/IP tasked with tracking MDROs. Percentage of facilities registered for VABA was evaluated at baseline, Follow-up 1, and Follow-up 2 (Figure 1). Secondary outcomes evaluated included self-reported knowledge, use of, and intentions to register for VABA through pre- and post-education surveys of target users. Barriers to and facilitators of adoption were also identified from guided interviews.

### Data collection

VABA registration was obtained from the MDRO Prevention Division. Data on facility characteristics were obtained from administrative records in the VA's Corporate Data Warehouse from fiscal years 2021–2022 (October 2020–September 2022; Figure 1). These characteristics included geographic region, complexity, and rates of VABA-eligible MDROs (MRSA, CRE, and VRE). The list of unique facilities for VABA registration analyses was obtained from VHA Support Service Center (VSSC) administrative records and the VA facility listing website (33).

Voluntary, identifiable (non-anonymous) pre- and post-education surveys were delivered via email using SurveyMonkey by the MDRO Prevention Division to a total of 651 target users across 121 facilities with active MPCs/IPs nationwide. Education session attendance was unknown. Therefore, surveys were sent to all known target users regardless of whether they attended the education session. (Supplementary Appendices 1, 2 include surveys.) Recipients included 283 MPCs, 61 IPs, and 307 individuals serving as both MPC and IP.

After randomization, facilities randomized to enhanced implementation were invited via email to participate in guided interviews. Up to one email reminder, one MS Teams message, and one phone call were sent to potential guided interview participants. If an MPC could not be reached at that facility, the implementation team identified and contacted the local facility's IP. Our purposive sampling strategy involved a limited pool of eligible key informants (i.e., target users at unregistered sites). Given this, guided interviews were conducted until recruitment attempts were exhausted rather than until thematic saturation was reached (34).

The guided interview guide (Supplementary Appendix 3) was developed by the research team using concepts from user-centered design and piloted in the Standard Implementation Period (35, 36). Participants were told that guided interviews would last a maximum of 30 min. Guided interviews were audio recorded and transcribed using MS Teams. Transcripts were uploaded into MAXQDA, a qualitative data management and analysis software program (VERBI Software, Berlin, Germany), for subsequent analysis.

### Data analysis

Pre- and post-education surveys were analyzed using descriptive statistics including frequencies and percentages. Facility-level VABA adoption (the percentage of facilities with at least one registrant for VABA) was compared between baseline and follow-up using McNemar's test. Facility complexity and geographic region were compared by facility registration status at baseline and Follow-up 1 using Chi-square tests. We obtained MDRO incidence-rate differences by registration status. Facility characteristics (complexity and geographic region) for standard vs. enhanced implementation groups of the cluster-randomized control implementation trial were assessed using Fisher's exact test to ensure no differences between groups. Follow-up 2 adoption rate was compared by implementation condition group using Fisher's exact tests. MDRO incidence-rate differences by implementation group were obtained.

Implementation groups were defined by assigned implementation condition in an intent-to-treat analysis, by whether the facility completed a guided interview in a second analysis, and by whether the facility received VABA coaching (i.e., not only participated in a guided interview, but agreed to hear more information and guidance about VABA during the guided interview and thereby “completed full external facilitation,” as we henceforth describe these facilities) in a *post-hoc* sensitivity analysis. In this analysis, all sites that did not complete full external facilitation were grouped together. This category therefore contained standard implementation sites, enhanced implementation sites that did not complete guided interviews, and an enhanced implementation site that completed a guided interview but declined additional information about VABA (and thus did not complete full external facilitation). We also examined differences in facility characteristics by guided interview completion status to identify any differences that may have potentially biased results.

VABA utilization is only available at the facility level. Therefore, for multi-hospital facilities, the highest-complexity hospitals with MDRO admissions during the evaluation rating were chosen for analyses. These hospitals likely weighed most heavily in their target users' VABA adoption decisions. All quantitative analyses were conducted using StataMP 17 (37). The threshold for statistical significance was set at *p* < 0.05 for all analyses involving inferential statistics.

Guided interview responses were coded using an inductive/deductive approach (38). Deductive codes were identified using the Systems Engineering Initiative for Patient Safety (SEIPS) 2.0 framework (39). SEIPS 2.0 specifies how work system elements influence “processes” (e.g., care processes, teamwork), and “outcomes.” Elements include “tools/technologies,” “tasks,” “persons,” “internal environment,” and “organization.” All work elements may interact with each other. See Supplementary Table S1 for the coding scheme. SEIPS is based on decades of healthcare research. SEIPS has been successfully applied to understanding the adoption and implementation of infection prevention measures and healthcare information technology, including systems used in VA to access information from other facilities (40–49). Our inductive approach allowed themes on how work-systems components interact and shape VABA adoption) to emerge (38, 50).

All transcripts were double-coded by trained qualitative researchers (CR, CCG, AMH) with disagreements resolved via consensus for 100% agreement. A matrix approach was used to examine intersecting codes and organize excerpts into themes for sensemaking purposes. We report on themes that were deemed well-represented within the data (i.e., that were reported by two or more facilities). Participants were not provided the opportunity to validate transcripts or findings.

## Results

### Pre- and post-education surveys

Demographics and responses of MPCs/IPs to pre- and post- survey questions are presented in Supplementary Table S2. 167 individuals across 116 VA facilities responded to the pre-education survey (representing 95.9% of the 121 facilities). VABA familiarity and use were low before the education session. Only 25.1% (*n* = 42) were familiar with VABA's previous iteration and had used it. Those who reportedly used VABA most often responded that they used VABA monthly (28.6%). Only 9.5% used VABA every day.

92 individuals across 80 VA facilities completed the post-education survey (facility response rate = 66.1%). Of these, 76 (81.7%) said they planned to register for VABA. Among the 16 participants who reported that they would not or were unsure whether they would register, the most common reason cited for not registering was that they already had a way to obtain the information provided by VABA (e.g., a commercially available tool; *n* = 11, 70.6%). Of the subset who planned to register for VABA (*n* = 76), 71.1% said they would use the feature to create custom email alerts.

### Facility characteristics and VABA adoption

Exclusions and participation in each component of standard and enhanced are displayed in Figure 1. Characteristics of 121 VABA-eligible facilities were compared by registration status at baseline and after standard implementation in Table 2. At baseline, facilities that were registered were higher complexity (*p*-value = 0.004) and had higher rates of VRE cases per 10,000 patients (*p*-value = 0.03) than those that were not registered. At Follow-up 1, there were significant geographic differences (*p* = 0.005) such that no unregistered facilities were from the Northeast, and unregistered facilities were more commonly from the South and West. Fiscal year 2022 MRSA and VRE rates were lower among facilities that had registered at Follow-up 1 (*p*-value < 0.001 for both) than those that had not, while CRE rates were higher (*p*-value = 0.04). The adoption rate was 40% at baseline and 75% at Follow-up 1. (*p*-value < 0.0001).

**Table 2:** Facility characteristics by VA bug alert registration at baseline and follow-up 1.

Variable	Registered for VABA* at baseline			*p*	Registered for VABA* at follow-up 1			*p*
	No, *N* = 73 (60.3)	Yes, *N* = 48 (39.7)	Total, *N* = 121 (100.0)		No, *N* = 31 (25.6)	Yes, *N* = 90 (74.3)	Total, *N* = 121 (100.0)	
Facility Complexity				**0** **.** **004**				0.20
High	40 (54.7)	39 (81.3)	79 (65.3)		24 (77.4)	55 (61.1)	79 (65.3)	
Medium	12 (16.4)	6 (12.5)	18 (14.9)		2 (6.5)	16 (17.8)	18 (14.9)	
Low	21 (28.8)	3 (6.3)	24 (19.8)		5 (16.1)	19 (21.1)	24 (19.8)	
U.S. Geographic Region				0.33				**0**.**005**
Midwest	13 (17.8)	12 (25.0)	25 (20.7)		7 (22.6)	18 (20.0)	25 (20.7)	
Northeast	16 (21.9)	9 (18.8)	25 (20.6)		0 (0.0)	25 (27.8)	25 (20.6)	
South	31 (42.5)	14 (29.2)	45 (37.2)		13 (41.9)	32 (35.6)	45 (37.2)	
West	13 (17.8)	13 (27.1)	26 (21.5)		11 (35.5)	15 (16.7)	26 (21.5)	
Rates per 10,000 patient admissions:**	2021				2022			
MRSA	7.8	8.2	8.0	0.10	9.2	7.8	8.2	**<0**.**001**
CRE	0.3	0.2	0.3	0.05	0.2	0.3	0.3	**0**.**04**
VRE	1.7	2.0	1.8	**0**.**03**	2.4	2.0	2.1	**<0**.**001**

* Facilities where at least one person is registered for VA Bug Alert.

** For these variables, three facilities were omitted as they had missing data due to transitioning to a new electronic medical record system, and numbers presented are incidence rates and *p*-values for incidence-rate differences; otherwise, numbers presented are *N* (%), *p*-value for Chi-Square.

Bold values indicate significance at the *p* < 0.05 level.

CRE, Carbapenem-resistant *Enterobacterales*; MRSA, Methicillin-resistant *Staphylococcus Aureus*; VABA, VA bug alert; VRE, Vancomycin-resistant *Enterococci.*

Of the 31 facilities that were not registered at Follow-up 1 and that had confirmed MPCs, 15 were randomized to enhanced implementation, and 16 were randomized to continued standard implementation. Of the 16 enhanced implementation facilities, 9 completed guided interviews. Of these, 8 facilities accepted additional information and coaching about VABA during the guided interviews. Thus, across both conditions, 8 facilities completed full external facilitation, and 23 did not.

### Adoption of VABA and facility characteristics by implementation condition

Table 3 displays facility characteristics and registration status at Follow-up 2 by assigned implementation condition of facilities (the intent-to-treat analysis) and whether they completed full external facilitation. There were no significant differences in facility complexity or geographic region by type of implementation assigned or received. However, VRE incidence rates per 10,000 patients were significantly higher among those assigned to standard implementation than enhanced implementation (*p*-value = 0.003). Furthermore, rates of CRE, VRE, and MRSA per 10,000 patients were significantly higher among the 23 facilities who did not complete full external facilitation (i.e., both participate in guided interviews *and* accepted coaching about VABA during said guided interviews) than the 8 who did (*p*-value < 0.001 for all).

**Table 3:** Facility characteristics and VA bug alert registrations by implementation Status.

Variable	Total, *N* = 31 (100.0)	Assigned Condition (Intent-to-Treat)p		*p*	Completed full external facilitation		*p*
		Standard, *N* = 15 (48.4)	Enhanced, *N* = 16 (51.6)		No, *N* = 23 (74.2)	Yes, *N* = 8 (25.8)	
Facility Registered* for VABA at Follow-up 2				0.48			**0.045**
No	14 (45.2)	8 (53.3)	6 (37.5)		13 (56.5)	1 (12.5)	
Yes	17 (54.8)	7 (46.7)	10 (62.5)		10 (43.5)	7 (87.5)	
Facility Complexity				0.56			1.00
High	24 (77.4)	11 (73.3)	13 (81.3)		17 (73.9)	7 (87.5)	
Medium	2 (6.45)	2 (13.3)	0 (0.00)		2 (8.7)	0 (0.0)	
Low	5 (16.1)	2 (13.3)	3 (18.8)		4 (17.4)	1 (12.5)	
U.S. Geographic Region				0.53			0.12
Midwest	7 (22.6)	3 (20.0)	4 (25.0)		6 (26.1)	1 (12.5)	
South	13 (41.9)	5 (33.3)	8 (50.0)		7 (30.4)	6 (75.0)	
West	11 (35.5)	7 (46.7)	4 (25.0)		10 (43.5)	1 (12.5)	
2022 Rates per 10,000 patient admissions of:**							
MRSA	9.2	9.2	9.3	0.77	9.8	7.3	**<0**.**001**
CRE	0.2	0.1	0.2	0.08	0.2	0.0	**<0**.**001**
VRE	2.41	2.8	2.1	**0**.**003**	2.7	1.6	**<0**.**001**

* Facilities where at least one person is registered for VABA.

** For these variables, numbers presented are incidence rates and *p*-values for incidence-rate differences; otherwise; *N* (%), *p*-value for two-sided Fisher’s exact test.

Bold values indicate *p* < 0.05.

CRE, Carbapenem-resistant *Enterobacterales*; MRSA, Methicillin-resistant Staphylococcus Aureus; VABA, VA bug alert; VRE, Vancomycin-resistant Enterococci.

At Follow-up 2, 89.2% of eligible facilities were registered for VABA (*n* = 107), which was a significant increase from baseline (*p*-value = 0.0001). Overall, 10 enhanced implementation facilities (62.5%) registered for VABA, compared to 7 standard implementation facilities (46.7%) (*p* = 0.48). A sensitivity analysis found that while 7 of the 8 facilities completing full external facilitation registered for VABA (87.5%), only 10 of the 23 facilities that did not complete full external facilitation registered for VABA (43.5%), such that completing full external facilitation was associated with significantly more frequent VABA registration (*p* = 0.045).

### Factors influencing adoption—guided interviews results

Guided interviews were completed by participants from 8 facilities (out of 13 eligible facilities in the enhanced implementation condition; response rate = 61.5%). Guided interviews lasted 20.5 min on average (between 12.1 to 29.0 min). Emergent themes centered around factors influencing VABA adoption including perceptions about VABA, organizational factors, and implementation activities (Table 4).

**Table 4:** Key themes and illustrative quotes regarding factors influencing VABA registration from guided interviews.

**Theme I: Individual perceptions about VABA's fit with end-user needs influenced VABA adoption**
1. Participant: “… if I set it up like for every, I don't know, four hours– I like to look at my stuff—I ideally- I like to look at the beginning of shift and the end of the shift. So, if there was something there, there would be an alert already ready for me, so I wouldn't have to look through everything; I would know to focus in on that, correct?”
Interviewer: “That is correct, right. At least how I—”
Participant: “I like– I like that.” Facility 12
2. “Interviewer: …and I think they just added C. Auris and VRE as well.
Participant: Perfect … Those would be helpful.” Facility 9
3. “Interviewer: And one thing that VA Bug Alert can do that some of the other methods you described might not be able to capture is that it'll notify you if a patient that’s MDRO-positive is coming in from another facility … and you can also set any notification settings for that type of thing to only let you know about cases coming in from other facilities. So, say if you already get quite [a large] amount of e-mail notifications just from [the Veterans Health Information Systems and Technology Architecture] … You could set alerts to only pertain to those to those outside cases and same for viewing their reports, too. You can also filter the reports to just focus on specific organisms or just focus on results for patients coming in from other facilities. So … I'll stop and just open it up to see if you have any thoughts about what I've told to you or how you think it might or might not fit into your workflow.
Participant: I like the aspect of getting the information of outside test results before someone's transferred over. So, knowing ahead of time.” Facility 13
4. “Well, the software is there, and I would be checking it frequently to see the result what’s on there. That's all we do, [non-VA software] and the lab—we go in Vista and check all our alerts and transfer them to CPRS and If there is an HAI we, do what we have to do on it. So, it would be the same [for VA Bug Alert].” Facility 6
5. “Anytime that I've used it [previous iteration of VA Bug Alert], it's been it's been accurate and fine, I just—because of our workflow— I guess we could switch our workflow and rely on the VA information first, but it just it fits into the way we do the our processes here quicker and easier, and then we use the VA Bug Alert to validate what we were finding.” Facility 9
“… I feel like some question(s) … could be shortened, because when you check review, it says, uh, then there’s small window, two consecutive small windows. I feel that could be shortened, …but each individual patient you click … that's kind of time-consuming, and then people get fatigue and not- like, like, me, if I have lots of things, I get distracted. I couldn't finish the list, so I felt that part could be shortened.” Facility 10
**Theme II. Organizational factors influenced VABA adoption.**
6. “Interviewer: I am curious as to whether you think the process of at least having alerts just on certain … organisms coming in from other facilities … would be helpful…
Participant: Yeah, I think it would be- would be very helpful. You know … we don't get a ton of people from other stations here in [Facility 9]. But we do every now and then. And then the number of them that would have an MDRO would be even less. So, the number of alerts we would get… I mean, I yeah, it would be helpful, if, again, for no other reason but to validate that we have on them here, or maybe we misread a, a culture report and didn't see, a resistance to a certain antibiotic that we needed to catch and then the VA Bug Alert would alert us to that MDRO that we may have missed, but, as I said earlier, we have not had that issue yet, and it’s always been nice to have it pop up. But yeah … I think being able to set it for only selected high consequence-type MDROs would be nice.” Facility 9
7. “I didn’t care for [VABA], it seemed to take a fair amount of time to load it, and then also I’m trying to compare—there’s a midnight CAB report we get as well. And the thing I don’t like about that report is that it specifically states, “do you want to review labs within the last 30 days?” Well, if I’m in the middle of the month or something and it's been weeks before I could get to reviewing your something, you can't go back to that last day you reviewed that report. So that's kind of a headache.” Facility 13
8. “**Well, can I ask one question first because we're switching to [new electronic health record system], so is this even gonna matter?** …The reason why and we all feel this way, we've discussed prior to this meeting the reason why we've never used the Bug Alert system is because we have such a good relationship with our lab. They call us, they e-mail us. Our reports are generated on a daily basis … To my knowledge, we've never missed something, and in addition to that, our providers stop in the lab every day. So sometimes they know if something is cooking before we even know, so having said that, that’s why we've never jumped on board and I'm just being honest, but also trying to be respectful, so I guess…**I don't necessarily… feel the need to hear anything further until [new electronic health record system] is live, because if we start having an issue after [new electronic health record system] is live, then I think I would want to hear what the VA bug alert could do for us.**” Facility 8
9. “I- I got the I got your e-mail, and I am gonna look at the slides and I will talk to [Quality Chief?] and see what she thinks about it and see what she knows about it, and if she wants me to sign up for it. I'll be happy to do it, but I do need to talk to her before I sign up for it.” Facility 12
10. “Interviewer: I take it you haven't tried signing up for VA Bug Alert?
Participant: No, I had not, ‘cause I also, of course wanted to get authorization from my supervisor and I did inform her about it and so I hadn't quite gotten a –I didn't wanna move too far in advance without letting them know, “Hey, this is something you know, is this something that we wanna get involved in our or take part in, so– and she’s out right now, so… Hopefully I'll get a response from her soon to take a little bit more– get more information.” Facility 2
11. “I know I was on one of the meetings when they were discussed, they were– right when they were discussing and getting some information, giving information out about it. But it was a while ago and I don't remember much of it. The last two times they had a meeting, unfortunately, I was out ill and didn't get to get the additional information about that and had not had time to go back on to the SharePoint to revisit it. With our numbers going the way they are, it's been pretty busy, unfortunately.” Facility 2
See also quote 13.
12. “Interviewer: …do you have any recommendations for things the MDRO program office could do better in order to increase utilization of the tool?
Participant: Just educate, like you're doing now. [Laughs softly] Just educate more on what it is, you know, I think I came in—I know I didn't go over this. I didn't go to this education because I wasn't here long enough, and I've been getting bits and pieces of it, but I didn't really understand what it was. So yeah, it looks like a handy program.” Facility 12
13. (Shortly after quotes 11 and 12): “So, I was kind of I was pleased to see that you guys inquired to do an interview because I definitely want to get more information so we can make a well-informed decision and really be up to par on what’s going on.” Facility 2.
14. “Participant: You said this is something that VAs are already using; when you tell us we would subscribe, is this something … that we currently own, or you're asking us to purchase?”
Interviewer: “Oh no, no, no. It is completely free…” Facility 1
15. “Participant: And the only other question that comes to mind, because I'm not used to working in the VA system—this is not gonna cost the hospital, right?
Interviewer: That’s correct. This is made by VA, for VA, and it is completely free.
Participant: OK, I don't want to sign up for something that's gonna get me in a bind or something like that, so, I have to ask that question.” Facility 12
16. “Interviewer: So in that case then the main component or piece of information that the- VA Bug Alert might be able to contribute beyond that existing system you're describing, is that it'll it can let you know if you're getting a transfer coming in from another facility that’s MDRO-positive, so they can be placed in contact precaution[s] sooner rather than later.
Participant: Yes, that sounds great, because I don't think that that actual log does that, so that would be great.” Facility 4.

Systems Engineering Initiative for Patient Safety 2.0 constructs are italicized.

**Theme I: Individual perceptions about VABA**'s **fit with end-user needs** (e.g., preferences, intended tasks, existing processes or workflows, and usability needs) **influenced adoption**. For example, one participant liked that they could customize alert settings to their timing preferences (e.g., to receive them at start of shift; quote 1). Another user described alerts of select organisms (quote 2) as being useful. Yet another user described liking that VABA provided alerts of cases coming from other facilities ahead of time (i.e., a useful feature of VABA; quote 3). VABA adoption also appeared to be supported to the perceived extent that VABA use fits in users' existing processes or workflows (quotes 4 and 5). Conversely, viewing VABA as time-consuming or difficult to use (i.e., low usability) seemed to inhibit VABA adoption (quote 6).

**Theme II: Organizational factors influenced adoption.** Some organizational factors appeared to influence perceived fit of VABA with users' needs; for example, the facility's volume of MDRO cases that could be identified by VABA as opposed to other available tools (Quote 7). Similarly, VABA adoption was also impeded by perceived incompatibility with other technologies provided by the organization (quotes 8 and 9). Other organizational influenced adoption outside of directly shaping perceptions about VABA's fit with their needs. For instance, some target users reported needing to obtain supervisor permission before they could adopt VABA after coaching (quotes 10 and 11). Staffing challenges such as absences, turnover, or high workload made it difficult to attend education sessions (quotes 12 and 13) or review the presentation slides after the session (quote 12).

**Theme III: Implementation activities (e.g., external facilitation) appeared to influence adoption** and hinge on the extent to which they could compensate for organizational barriers or highlight relevant positive attributes of VABA. Specifically, quotes 13 and 14 suggest that the one-on-one guided interview format of external facilitation may have helped compensate for these participants' aforementioned inability to make the education session (quotes 12 and 13). Quotes 15 and 16 show external facilitators pointing out that VABA is free within VA (reportedly an important precondition of VABA adoption according to quote 16). Furthermore, participants responded favorably to information about VABA attributes that fit their needs. Such attributes included VABA's interfacility transfer tracking abilities (quote 17) and alert customizability (quotes 1 and 7).

## Discussion

### Key findings

Across 121 VA facilities, VABA adoption was low at baseline. Registration increased after education, where three-quarters adopted VABA. Enhanced implementation strategies were associated with improved VABA adoption compared to standard implementation (Follow-up 2). However, this finding was specific to the coaching component of external facilitation in a *post-hoc* sensitivity analysis. This may have been because the coaching component of external facilitation may have compensated for the effects of key organizational influences such as staffing (that constrained the reach of education sessions). Coaching also appeared to convey important adoption-relevant information, for instance, about VABA's fit with user needs. Thus, our efforts appeared largely successful for increasing VABA adoption.

Target users' perceptions that VABA fit their preferences and needs were related to adoption, as particularly illustrated by guided interview findings. Furthermore, survey respondents who reportedly did not intend to adopt VABA often indicated that they already had a method of obtaining the information provided by VABA. In other words, for this subset of participants, VABA did not have additional perceived usefulness beyond that of other tools they were already using. These findings are consistent with prior evidence illustrating the importance of perceived usefulness and usability on technology use (51–54). VABA's compatibility with existing processes was another important determinant of adoption. Similarly, other studies have found that implementation of antimicrobial stewardship interventions is linked to the interventions' fit or compatibility with existing processes (55–57). Our findings also echo work outlining the role of innovation adaptability, complexity, design, relative advantage over alternative tools, and inner setting compatibility in implementation (58).

Organizational factors also shaped VABA adoption. They appeared to do so in part by limiting the reach or impact of implementation activities. For instance, VABA adoption was constrained by the perceived need to obtain supervisor permission to register, even following external facilitation. Attendance of large-group education sessions was impaired by staffing challenges at unregistered facilities, as previously described in a past systematic review (26).

Implementation activities shaped adoption. The significant increase in registration from baseline to after follow-up 1 is concordant with past reports demonstrating the effectiveness of provider training as an implementation strategy (59, 60). We also found that external facilitation (specifically, the coaching component of guided interviews) increased registration. This finding also partially echo past work demonstrating the effectiveness of external facilitation (9, 10). The success of implementation activities for promoting adoption also appeared to hinge on the extent to which they could influence target users' perceptions, also consistent with prior evaluations (61). External facilitation highlighting VABA's customizable features (i.e., adaptability) appeared effective for fostering adoption. This is perhaps because external facilitation demonstrated that VABA can meet end-user preferences and needs, as illustrated by the exchange in Table 4, quote 1.

Implementation activities' success also seemed to depend on the extent to which they could surmount organizational barriers to implementation. External facilitation provided additional support for staff who were unable to attend education sessions due to staffing constraints, also consistent with a past systematic review (26). Strategies more commonly recommended to reduce challenges with available resources, such as obtaining more funding to hire additional staff, may be infeasible in some cases (58, 62). Approaches such as external facilitation may be particularly helpful in such instances by setting aside time in busy target users' schedules for one-on-one interaction.

### Implications for future innovation design and implementation efforts

Findings emphasize the need to identify and address organizational barriers and end user needs or preferences when designing and implementing technological interventions (26, 62). Our results also illustrate the importance of engaging key stakeholders for identifying these key considerations (12). Tailoring of implementation to local context can be guided by stakeholder feedback (17–20, 25).

Our findings and expert recommendations also suggest which implementation strategies may be most useful to combat specific barriers for future innovation design and implementation efforts. For instance, stakeholder engagement should involve individuals beyond target users, such as their supervisors. This strategy can help obtain clear support and permission for intervention use. This suggestion is consistent with that of Miake-Lye and colleagues' suggestion to promote “visibility with multi-level leadership” of late adopters (26). It also aligns with experts' endorsement of informing local opinion leaders to address issues with key stakeholders and opinion leaders (58, 62).

When perceived compatibility of intervention is low, it may be beneficial to promote adaptability and implement cyclical small tests of change (25, 49). A systematic review highlighted two strategies: (1) enhancing the adaptability of innovations and (2) refining them based on feedback from trials with late adopters. These strategies may increase an innovation's appeal or perceived advantage relative to alternative tools (26, 58). Additionally, one project that used the SEIPS 2.0 model to evaluate a tool designed to convey safety-related information about patients from outside facilities (similar to the current project) found that tool usage improved when its fit with the intended task (i.e., usefulness) was enhanced (49). User-centered design can guide tailoring and (re)design promote implementation (35, 36, 58, 63–65). It can do so by incorporating end-user perspectives into intervention design and implementation to ensure user needs are met (35, 36, 58, 63–65). More studies should explore this possibility (66).

### Strengths and limitations

This project has several strengths. Our adaptive trial design, multiple periods of data collection, and novel guided interview approach allowed us to refine our implementation strategies in response to differences in beliefs across users and shifting contexts over time. Our semi-structured guided interview included suggested facilitator responses to comments from interview participants promoted standardization of the provision and measurement of often “black box” external facilitation processes (67) and helped to address complexities associated with navigating dual roles of interviewer and external facilitator. To our knowledge, this is the first report describing a formalized method and guide for collecting data on barriers and facilitators, providing external facilitation support, and collecting data on initial reactions to external facilitation in a single interview. This method thus provides structure to the interactive problem-solving processes of external facilitation (15).

Our analysis included guided interview text comprising VABA guidance from external facilitators/interviewers in addition to participant responses. This approach allowed us to extract the content conveyed during the guided interviews that appeared to promote adoption intentions. Furthermore, access to VABA utilization metrics allowed us to measure behavior directly, rather than intentions reported in surveys and guided interviews alone.

We used the SEIPS 2.0 framework, which connects work system inputs with processes and outcomes and allows for interactions between work system elements. This in turn gives flexibility for understanding and contextualizing implementation of technological interventions. We were thus able to identify relationships between technology adoption and other work systems elements from the ground up without pre-conceived notions. These relationships correspond to prior implementation science constructs despite not looking for those constructs a-priori. This correspondence provides particularly compelling evidence for the role of these constructs in adoption.

Despite the strengths of this project, it also had some limitations. Because educational module attendance was unavailable, we were unable to calculate survey response rates among attendees. Because VABA utilization metrics were only available at the integrated facility/healthcare system level, in which some facilities comprise multiple medical centers, we were unable to assess the role of implementation strategies and possible covariates on registration of each medical center (68). Furthermore, VABA registrants may have included individuals using VABA for administrative or reporting purposes rather than frontline MDRO prevention efforts. Thus, our registration numbers may include others besides target users. The ineligibility of some facilities for enhanced implementation and VABA use was discovered after randomization and implementation began. Thus, the analyses include some facilities not eligible for VABA (69, 70).

The small sample of facilities participating in the trial compromised the statistical power to detect effects of enhanced implementation. Furthermore, higher VABA adoption among sites that received enhanced implementation as intended (which included the guided interviews) as compared to those who did not may have been influenced by the lower MDRO rates rather than the external facilitation itself. Thus, evaluation of the effectiveness of external facilitation efforts from this study must be interpreted with caution, as resource-intensive strategies such as external facilitation may not always be merited. This may particularly be true in cases such as the current initiative, in which late adopters were scarce.

The scarcity of non-adopters attests to the success of the education session and tailoring of the tool based on prior target user surveys. This latter finding lends further credence to our recommendations regarding the importance of tailoring design based on user input. However, because all sites received standard implementation at a minimum, we cannot rule out the possibility that history effects, such as decreased competing priorities following the COVID-19 pandemic, drove increased registration from baseline. Finally, the increased VABA use from baseline in response to standard and enhanced implementation may be due to prolonged exposure to implementation efforts (i.e., time) rather than the merits of any one implementation strategy (e.g., internal facilitation).

## Conclusions

Overall, design tailoring and adaptation, education, and facilitation successfully promoted adoption of a MDRO alert tool in the VHA. These strategies were particularly successful to the extent that they addressed organizational factors and shaped end-user perceptions regarding the usability and utility of VABA. Future work includes in-depth analysis of internal facilitation processes. We will also continue to refine VABA design, implementation activities, and evaluation methods based on current findings and participant recommendations in collaboration with the MDRO Prevention Division. The goal of these activities will be to increase and sustain VABA use among registrants. Finally, we will evaluate the effects of VABA implementation and use on clinical processes (e.g., timeliness of isolation/contact precautions) and patient care outcomes (e.g., MDRO transmission).

## Data Availability

The datasets presented in this article are not readily available because we cannot directly share underlying data as it would compromise participants' anonymity, and permissions from VA are needed to obtain the data. We are committed to collaborating and sharing these data to maximize their value to improve Veterans and others' health and health care, to the greatest degree consistent with current Department of Veterans Affairs regulations and policy. We can provide access to the programming code used to conduct quantitative analyses. Requests to access the datasets should be directed to cara.ray@va.gov.
