# Supplementary material for: Standard vs. enhanced implementation strategies to increase adoption of a multidrug-resistant organism alert tool: a cluster randomized trial
Source: Front Health Serv. 2025 Sep 18;5:1566454. doi: 10.3389/frhs.2025.1566454 (PMC12488722; doi:10.3389/frhs.2025.1566454)
Supplement: Supplementary file 1 [file Table1.docx]

# **Supplemental Table 1: Code definitions.**

| Codes | Definition (refine as needed following review of a first few questions) |
| --- | --- |
| Barrier* | Barrier to VABA use; Barrier to patient care (including MDRO/infection prevention) |
| Task | A thing that is done or needs to be done From SEIPS: Variety of tasks, Job content, challenge and utilization of skills Autonomy, job control and participation Job demands (e.g., workload, time pressure, cognitive load, need for attention) |
| Person | Characteristics/aspects of people - education, knowledge, experience, expertise, motivation, needs, psychological characteristics, physical characteristics |
| Tools/technology | From SEIPS: Various information technologies: electronic health record, computerized provider order entry and bar coding medication administration Medical devices Other technologies and tools Human factors characteristics of technologies and tools (e.g. usability) Intervention characteristic: relative advantage (e.g. using other tools instead), compatibility/complexity (if there wind up being issues with [new electronic medical record system], tool being difficult to use or alert content/frequency not being customizable enough) |
| Background info* | The interviewee's role/position at their facility and how long they have occupied that position |
| Organization | Original definition from SEIPS: teamwork, coordination, collaboration, communication, organizational culture & patient safety culture, Work schedules, Social relationships, Supervisory and management style, Performance evaluation, rewards and incentives New-ish sub-constructs generated by team: resources (tools, personnel, e.g. whether they have an ID physician as a hospital epidemiologist, policies) structure/procedures (in this case, for things like infection control -- whether they have a team available or if it's just them) Inner setting (from CFIR): Readiness for implementation & Access to knowledge & information (e.g., people not knowing what the tool is or how to sign up due to turnover) |
| Process | Care processes  Other processes: information flow, purchasing, maintenance, cleaning  Process improvement activities |
| Outcomes | Patient Outcomes: Patient safety Quality of care Employee and organizational Outcomes: job satisfaction and other attitudes Job stress and burnout Employee safety and health Turnover |
| Codes | Definition (refine as needed following review of a first few questions) |
| Recommendations* | Responses to question about what tools/methods would be helpful for MDRO prevention if resources weren't an issue (even if there were no recommendations made), and any other recommendations for the MDRO Prevention Division or regarding VABA. |
| Questions Asked/ Information Shared/ External Facilitation* | Subcoding/analyzing this code as external facilitation/information provided by interviewer as data. |

*New code; otherwise, codes originated from the Systems Engineering Initiative for Patient Safety 2.0 framework.
