# Supplementary material for: Standard vs. enhanced implementation strategies to increase adoption of a multidrug-resistant organism alert tool: a cluster randomized trial
Source: Front Health Serv. 2025 Sep 18;5:1566454. doi: 10.3389/frhs.2025.1566454 (PMC12488722; doi:10.3389/frhs.2025.1566454)
Supplement: Supplementary file 2 [file Table2.docx]

Supplemental Table 2. Characteristics and responses of Survey Participants Pre- and Post- Education about VA Bug Alert

|  | Pre-Survey (N=168), No. (%) | Post-Survey (N=92),  No. (%) |
| --- | --- | --- |
| Role |  |  |
| IP | 50 (29.9) | 37 (43.0) |
| MPC | 58 (34.7) | 37 (43.0) |
| IP/MPC | 59 (35.3) | 18 (20.9) |
| Geographic region |  |  |
| Northeast | 37 (22.2) | 20 (21.7) |
| South | 62 (37.1) | 37 (40.2) |
| Midwest | 39 (23.4) | 9 (28.3) |
| West | 29 (17.4) | 26 (9.8) |
| Are you familiar with the VA Bug Alert (formally called Inpatient Pathogen Tracker)? |  |  |
| Yes, I am familiar with it, and I have used it. | 42 (25.1) | n/a |
| Yes, I am familiar with it, but I have not used it. | 59 (35.3) | n/a |
| No | 66 (39.5) | n/a |
| How often do you use the VA Bug Alert? (Among those who said they used it; n=42) |  |  |
| Not applicable, I don't use it. | 5 (11.9) | n/a |
| For reports only | 11 (26.2) | n/a |
| Weekly | 10 (23.8) | n/a |
| Monthly | 12 (28.6) | n/a |
| Every day | 4 (9.5) | n/a |
| I feel comfortable executing the steps to register for VA Bug Alert. |  |  |
| Strongly Agree | n/a | 33 (35.9) |
| Agree | n/a | 36 (39.1) |
| Neutral | n/a | 17 (18.5) |
| Disagree | n/a | 4 (4.3) |
| Strongly Disagree | n/a | 2 (2.2) |
| VA Bug Alert will be a useful tool for me. |  |  |
| Strongly Agree | n/a | 22 (23.9) |
| Agree | n/a | 40 (43.5) |
| Neutral | n/a | 23 (25) |
| Disagree | n/a | 5 (5.4) |
| Strongly Disagree | n/a | 2 (2.2) |
| I plan on signing up for VA Bug Alert |  |  |
| Yes | n/a | 76 (82.6) |
| Unsure | n/a | 12 (13) |
| No | n/a | 4 (4.3) |
| Indicate why you are unsure or will not sign up for the VA Bug Alert (select all that apply) (n=16) |  |  |
|  | Pre-Survey (N=168), No. (%) | Post-Survey (N=92),  No. (%) |
| I already have a way to obtain this information. | n/a | 12 (75.0) |
| Other (Please specify) | n/a | 5 (31.3) |
| The tool does not include all relevant MDROs. | n/a | 2 (12.5) |
| I do not feel the tool is relevant or useful to my current role. | n/a | 2 (12.5) |
| Does not track non-VA transfers. | n/a | 1 (6.3) |
| Very low incidence of relevant MDROs. | n/a | 2 (12.5) |
| Aspects of the tool responders plan to use (n=76) |  |  |
| Custom Email Alerts | n/a | 54 (71.1) |
| Review Patient Status | n/a | 53 (69.7) |
| Search Function | n/a | 46 (60.5) |
| Review Discharge Reports | n/a | 32 (42.1) |
| Other | n/a | 5 (6.6) |

Note: n/a: Not applicable, indicating the question was not included on the survey.
