# Supplementary material for: Standard vs. enhanced implementation strategies to increase adoption of a multidrug-resistant organism alert tool: a cluster randomized trial
Source: Front Health Serv. 2025 Sep 18;5:1566454. doi: 10.3389/frhs.2025.1566454 (PMC12488722; doi:10.3389/frhs.2025.1566454)
Supplement: Supplementary file 3 [file Supplementaryfile1.docx]

# Welcome to the Implementation of VA Bug Alert Project!

As of June 1, 77% of VA facilities with Infection Preventionists (IPs) and MDRO Program Coordinators (MPCs) have at least one registered VA Bug Alert user.

You are receiving this message because 1) you are at a facility that **does not have any registered users** of the VA Bug Alert and 2) your facility has been selected to receive **enhanced implementation** of the tool from the Combating Antimicrobial Resistance through Rapid Implementation of Available Guidelines and Evidence (CARRIAGE) II Quality Enhancement Research Initiative (QUERI) program.

In the next few weeks, someone from the CARRIAGE II team will contact you about scheduling an interview to learn more about your thoughts on VA Bug Alert. In addition, you will also receive regular (every 1-3 months) emails with VA Bug Alert facts and tips as indicated below.

**By using VA Bug Alert, you can…**

- find and review all inpatient cases of select urgent & serious MDROs at your facility in a single spot
- receive real-time email alerts of new MDRO cases – **even those coming from other VA facilities**
- customize which MDROs you receive alerts from and how often.

These features promote timely MDRO identification and management.

- **To sign up for VA Bug Alert, click** [**here**](https://forms.office.com/g/S1dZGFX3F1)**.**
- **Having trouble signing up? Contact** [**VHASLCBugAlertsSupport@va.gov**](mailto:VHASLCBugAlertsSupport@va.gov)**.**
- **New to VA Infection/MDRO prevention?** Request local PHI/PII access if you

have not already done so [here](http://ipec.vssc.med.va.gov/IC/Pages/Permissions.aspx).

- **For more information about VA Bug Alert,** check out the [User Guide](http://ipec.vssc.med.va.gov/IC/Documents/VABA%20User%20Guide.pdf) and

[education slides](https://dvagov.sharepoint.com/:b:/r/sites/VHATS/getting2zero/National%20MPC%20Call%20Minutes/2022/MPC%20Call_2022_04_19_VA%20Bug%20Alert_(VABA).pdf?csf=1&web=1&e=OjtaR2).

- **For questions about your involvement in the CARRIAGE II VA Bug Alert Implementation Project** contact Cara Ray at **Cara.Ray@va.gov**.
