# Supplementary material for: Standard vs. enhanced implementation strategies to increase adoption of a multidrug-resistant organism alert tool: a cluster randomized trial
Source: Front Health Serv. 2025 Sep 18;5:1566454. doi: 10.3389/frhs.2025.1566454 (PMC12488722; doi:10.3389/frhs.2025.1566454)
Supplement: Supplementary file 4 [file Supplementaryfile2.docx]

**Pre-Education Questions**

1. What is your role in infection control/prevention?
   1. MDRO Prevention Coordinator (MPC)
   2. Infection Preventionist (IP)
   3. Both
2. Are you familiar with the VA Bug Alert (formally called Inpatient Pathogen Tracker)? [TECHNOLOGY TOOLS] Y/N
   1. No
   2. Yes, I am familiar with it but I have not used it
   3. Yes, I am familiar with it and I have used it
3. How often do you use the VA Bug Alert?
   1. Every day
   2. At least once a week
   3. Monthly
   4. For reports only
   5. Not applicable, I don’t use it
4. How many IPs are at your facility?
   1. Zero
   2. One
   3. Two
   4. Three
   5. Four
   6. Five
   7. Six
   8. Seven
   9. Eight
   10. Nine
5. How many MPCs are at your facility?
   1. Zero
   2. One
   3. Two
   4. Three
   5. Four
   6. Five
6. How many both, IPs and MPCs?
   1. Zero
   2. One
   3. Two
   4. Three
   5. Four
   6. Five
   7. Six
   8. Seven
   9. Eight
   10. Nine
