# Supplementary material for: Standard vs. enhanced implementation strategies to increase adoption of a multidrug-resistant organism alert tool: a cluster randomized trial
Source: Front Health Serv. 2025 Sep 18;5:1566454. doi: 10.3389/frhs.2025.1566454 (PMC12488722; doi:10.3389/frhs.2025.1566454)
Supplement: Supplementary file 5 [file Supplementaryfile3.docx]

**Post-Education Questions-- Now that you’ve participated in VA Bug Alert training…**

1. Did you attend the April 19^th^ MDRO Program Coordinator Call or April 27^th^ Infection Prevention Call that went over the VA Bug Alert Presentation?
   1. Yes [go to question 2]
   2. No [end survey]
2. I feel comfortable executing the steps to register for VA Bug Alert. [PERSON]
   1. Strongly Agree
   2. Agree
   3. Neutral
   4. Disagree
   5. Strongly Disagree
3. VA Bug Alert will be a useful tool for me (e.g., will be using, relevant to your job, etc.)
   1. Strongly Agree
   2. Agree
   3. Neutral
   4. Disagree
   5. Strongly Disagree
4. I plan on signing up for VA Bug Alert. [PERSON]
   1. Yes
   2. No
   3. Unsure

If you selected “Unsure” or “No,” please indicate why (select all that apply):

1. The tool doesn’t include all relevant MDROs
   - 1. If so, please list which MDROs: ___________________________________________________________
2. I don’t feel the tool is relevant or useful to my current role.
3. Doesn’t track non-VA transfers
4. I already have an easy way to obtain this information
   - 1. If so, please list the tools/methods you use________________________________________________________
5. Very low incidence of relevant MDROs
6. Other: __________________________________________________________________
7. What aspects of this tool do you plan to use (check all that apply)? [PERSON]
   1. Search Function
   2. Custom Email Alerts
   3. Review Patient Status
   4. Review Discharge Reports
   5. Other: ________________
