# Supplementary material for: Standard vs. enhanced implementation strategies to increase adoption of a multidrug-resistant organism alert tool: a cluster randomized trial
Source: Front Health Serv. 2025 Sep 18;5:1566454. doi: 10.3389/frhs.2025.1566454 (PMC12488722; doi:10.3389/frhs.2025.1566454)
Supplement: Supplementary file 6 [file Supplementaryfile4.docx]

Thank you for agreeing to talk with us today. As you may be aware, the VA MDRO Program Office recently launched the VA Bug Alert, or VABA (formerly known as the Inpatient Pathogen Tracker), which was presented in education sessions on the April 2022 and January 2023 IP & MPC calls. Our goal with this interview is to better understand your needs and goals as well as your thoughts about VA Bug Alert to improve VA Bug Alert’s usability. Interviews will last approximately 30 minutes and will focus on how VA Bug Alert might be able to meet your individual MDRO prevention needs. If there is any question you do not wish to answer, we can skip it. You can ask to end the interview at any time. Your responses will be anonymous. Aggregate, de-identified information will be shared with the MDRO Program Office to inform them of potential ways to improve VA Bug Alert and increase its use. With your permission, we may offer some information about VA Bug Alert’s features and how VA Bug Alert might be most useful to you. We hope that the findings from this project will be used to improve MDRO prevention and quality of care for all Veterans, in particular, decreasing the time in which MDRO positive Veterans are identified and placed into appropriate contact precautions. To ensure that we accurately capture your responses, we would like to audio-record the interview. Do we have your permission to audio-record our conversation today?

**If no:** That’s fine. We will take detailed notes while we talk.

**If yes**: Thank you again for your participation. I will now begin the recorder. **[START AUDIO RECORDER]**

This is to advise you that I have turned the recorder on. To document your recorded permission, I need to ask you again if you agree to have this conversation recorded. Do you agree? <Wait for “YES” reply from participant> Thank you. I will now begin with the interview questions.

First off, we would like to ask a few questions about your background.

1. (Refer to background information from Natalie) Just to confirm, you are an [IP/MPC] from [X facility], correct?
2. How long have you been the [IP/MPC] at your facility?
3. We are going to be talking about MDROs. We are particularly focused on CRE, MRSA, VRE, C. Auris, and Carbapenem-Resistant Acinetobacter or CRAB. Can you describe [/walk me through] your process for identifying patients admitted to your facility with MDROs?
   1. Are there any advantages or disadvantages to this approach
   2. What could be useful to overcome those disadvantages?
   3. Are there any other barriers or facilitators you would like to mention?

Now, we are going to ask some questions specifically about VA Bug Alert.

1. Can you tell me what you’ve heard about VA Bug Alert?
   1. Did you attend the training on (*for MPCs:*) April 19, 2022? Did you attend the second training on January 17 2023? (*for IPs*:) Did you attend the training on April 24, 2022? Did you attend the second training in January 2023?
   2. [If yes:] Did you find the trainings helpful for knowing how to use VABA?
   3. [*If not:*] did you see the slides, or did you plan to?

[*If not, probe for a reason if they do not offer one initially; listen for, among other things, potential ways for the MDRO Program Office to consider improving how they share information if applicable]*

1. What are your thoughts or impressions about VA Bug Alert?
   1. [*Probe:*] Is the website easy enough to navigate? (We are interested in letting the MDRO program office know what about VABA is or is not easy to use, so if you would like, one of us could pull up the website now if you would like to walk us through exactly how you would use it.) (Probe: Is there anything in particular you like about the site or find easy to use or navigate? Is there anything you dislike or find difficult to use or navigate?) What would help you use it or increase your use of it?
2. I see you (or someone at your station) is signed up for X alerts [plug in from audit and feedback report] and accessed the report X time(s) directly from the VABA website. Is that an accurate description of your understanding of how you use VA Bug Alert?

[**If No,** ask them to clarify how they use it.]

Is that how you intended to set it up and use it? Are you satisfied with how you are using it, or were you hoping to use it differently/more often?

**[If they are unsatisfied with their current use/would like to use it differently,** ask them how they meant to or would like to set it up. **If it sounds like they had trouble/didn’t know how to do what they would like,** offer to send them the user guide and support email.]

**[If they are satisfied with how they use it, move to the next section]**

**[For those not signed up for alerts:]**

1. Is there a particular reason why you did not subscribe to alerts? [Listen for the following examples and probe with some of them if the participant is not forthcoming with a response:] Did you…
   1. not know that you could or not know how
   2. **try to sign up but were unsuccessful**
   3. think that the process seemed difficult/time consuming
   4. not think they were useful/necessary? If so, why? Do you…
      1. Not have many cases of the organisms in question?
      2. Use other tools/methods (if so, which)?
   5. Do you need to obtain someone’s permission for whether to use VABA/ is someone else responsible for the decision to use VABA?
   6. Other reason (if so, what?)

[**if they did not know they could, did not know how, or thought the process seemed difficult,** offer them more info [i.e., a brief description of the features or offer to connect them with the support team or send them the user guide.] Probe for ways they think the ~~customization~~ process of setting up alerts could be made easier.

[**If they say they did not think they needed them,** probe for why not. Offer guidance on how the alerts may be able to make their job easier or improve clinical process outcomes as applicable, e.g., alerts from only other stations if they use Theradoc; alerts for organisms they have high rates of.**]**

**[for those who tried to but were unsuccessful:** [Ask about what went wrong, e.g., if they had difficulties navigating the site or if they received some type of error message. Listen for the following examples and probe with some of them if the participant is not forthcoming with a response:] Did you...

1. Have difficulties navigating the website
2. Not know which settings to select or how
3. Get some type of error message
4. Think the process was taking too long
5. Get interrupted before you could finish
6. Other

[**Ask if they are utilizing the steps in the User Guide and if they know how to report a problem using the VABA support email linked several places. Offer to send this information to them if needed. Then go to the section “For all participants”.]**

**[*For those signed up for alerts*]:**

1. Is there a reason why you chose those organisms (e.g., as opposed to including more pathogens ~~or receiving more frequent alerts~~)? [probe or listen for whether they actually meant to subscribe to those alerts]
   1. How was the process of subscribing to alerts? Was it easy? Was it difficult?
   2. What is your process for tracking and addressing pathogens you chose not to be alerted to?

[*Offer guidance on how VABA may be able to make their job easier or improve clinical process outcomes as applicable; e.g., if they are not signed up for alerts for which they have high incidence.]*

**[*If the participant says they only subscribed to default alerts*]** Did you try to customize alerts?

**[*If they tried to but were unsuccessful*:** Ask about what went wrong, e.g., if they had difficulties navigating the site or if they received some type of error message. Listen for the following examples and probe with some of them if the participant is not forthcoming with a response:] Did you...

1. Have difficulties navigating the website
2. Not know which settings to select or how
3. Get some type of error message?
4. Think the process was taking too long
5. Get interrupted before you could finish, or
6. Other

[Ask if they are utilizing the steps in the User Guide and if they know how to report a problem using the VABA email linked several places. Offer to send them this information. Then go to the “for all participants” section.]

**[If they did not:]** Why not? [Listen for the following; if they are not forthcoming with a response, probe:] Did you...

1. not know that you could
2. not know how
3. think that the process seemed difficult/time consuming
4. not think they were useful/necessary? If so, why? Do you…
   1. Not have many cases of the organisms in question?
   2. Use other tools/methods (if so, which)?
5. Other reason (if so, what?)

[***if they did not know they could, did not know how, or thought the process seemed difficult,*** *offer to put them in touch with the support team. Probe for ways they think the customization process could be made easier.]*

***[If they say they are fine with the default settings or that changing the settings would not be helpful,*** *ask about any organisms of which they have high rates that they may want to subscribe to, move to the next question.*]

1. What do you usually do when you receive a notification from VA Bug Alert of an MDRO+ case? [Probe:] Can you walk me through your workflow/what you typically do/your process once you’ve received a VA Bug Alert notification?

***[For all participants:]***

1. When you become aware of a new MDRO, do you pull up the report on the VA Bug Alert website?

[Probe:] was the process of pulling up the report easy/difficult?

1. Is there a particular reason why you haven’t pulled up the report [more than once/twice- insert from A&F report]? [Listen for the following; if they are not forthcoming with a response, probe:] Did you...
2. Not know that you could
3. Not know how to get to the report/ have difficulties navigating the website
4. Think that the process seemed difficult/time consuming
5. Not know which report settings to select or how
6. Try doing so but were unsuccessful/have difficulties
7. Not think it was useful or necessary to do so? If so, why? Do you…
   - 1. Not have many cases of the organisms in question?
     2. Use other tools (if so, which)?
8. Do you need to obtain someone’s permission for whether to use VABA/ is someone else responsible for the decision to use VABA?
9. Other (please specify)

| *[They give some indication that the question is not entirely applicable/ only hypothetical because they do not have any cases of the organisms for which they receive alerts]* | [*Skip to question about ways to improve bug tracker.*] |
| --- | --- |
| *[They say they review CPRS/Theradoc for details]* | [*Ask if they are aware that VABA reports show details about which patient(s) have which MDROs and, unlike other tools, tracks interfacility transfers. Offer user guide or education slides as needed.*] |
| [*They say the area (i.e., facility) they’re responsible for is so small they know who the notification is about without even needing to look*] | *[Ask how they track whether the patient has been attended to (e.g., placed on contact/isolation precautions). Then move to the question about ways to improve the bug tracker.]* |
| [*They say they don’t usually do much in response, or if they say that they do not view the report*] | [*Clarify their typical workflow for addressing key MDROs and what about that process works well for them if they haven’t discussed that already/ reiterate it.*    *Offer guidance, as applicable, on any ways the VA Bug Tracker might be able to address any challenges with MDRO prevention that they previously mentioned or are apparent from their A&F reports.]* |
| [They say they tried checking the report but were unsuccessful, did not know how, or found the process difficult] | [*Probe for which ways they found the tools helpful. Ask if they use the user guide or the support email. Offer user guide and/or education slides/ more info. Probe for ways the tool could be improved.]* |

1. How does VA Bug Alert fit within your workflow/daily activities? (or, for those not subscribed to alerts/haven’t accessed the report at all: Based on what information I shared about VA Bug Alert, how do you envision it fitting within your workflow/daily activities?) Why? [If they do not see it fitting into their workflow, probe:] Why not?
   1. [Probe:] Notifications are sent to your email—do you have time to check email? Would there be a better place to be notified?
   2. [Probe:] What about CPRS?
   3. [Follow-up question:] Do you remember receiving/ looking at the audit and feedback report(s)? [If yes] What did you make of the audit and feedback reports we sent you? Were they helpful for showing potential ways to use VA Bug Alert that might be of use?
      1. Did they change the way you used VABA after receiving it?
      2. Is there anything about the Audit and Feedback reports or how we shared them that you think could be improved?
2. Is there anything we could do that would get you to use VABA regularly?
3. Do you wish there were other functions? If so, what would those other functions be why would you like to use them?
4. Do you wish that it included different pathogens? If so, which?
5. If you could do anything – develop any sort of tool, system, or procedure – to facilitate communications and tracking regarding MDROs, and had all the means with which to do so, what would that look like? (*If additional clarification needed:* If you had a magic wand)
6. Is there anything you want the MDRO Program Office to know about the VA Bug Alert? Anything you think they could do to make it more useful or easier to use?
7. What recommendations/modifications, if any, would you make to improve the design/usability of VA Bug Alert?
8. Do you have any suggestions, tips, or ideas for things VA Bug Alert or the MDRO Program Office could do better in order to increase awareness and use? For example, do you have any other ideas for how the communications or trainings regarding VABA could be improved to encourage more people to use it?
9. What else should we know about your experience with the VA Bug Alert? Is there anything else you would like to talk about? Anything we should have asked you about? Is there anyone else at your facility we should talk to?

Thank you for participating! Your responses will be used to improve VA Bug Alert utilization.
